# The Nature of Illusions: A New Synthesis Based on Verifiability

**DOI:** 10.3389/fnhum.2022.875829

**Published:** 2022-05-31

**Authors:** Christopher W. Tyler

**Affiliations:** ^1^Division of Optometry, School of Health Sciences, City University of London, London, United Kingdom; ^2^Smith-Kettlewell Eye Research Institute, San Francisco, CA, United States

**Keywords:** illusions, perception, distortions, hallucinations, reality, Bayesian, adaptation

## Abstract

This overview discusses the nature of perceptual illusions with particular reference to the theory that illusions represent the operation of a sensory code for which there is no meaningful ground truth against which the illusory percepts can be compared, and therefore there are no illusions as such. This view corresponds to the Bayesian theory that “illusions” reflect unusual aspects of the core strategies of adapting to the natural world, again implying that illusions are simply an information processing characteristic. Instead, it is argued that a more meaningful approach to the field that we call illusions is the Ebbinghaus approach of comparing the illusory percept with a ground truth that is directly verifiable as aberrant by the observer in the domain of the illusory phenomenology (as opposed to relying on the authority of other experts). This concept of direct verifiability not only provides an operational definition of “illusion”; it also makes their interactive observation more effective and informative as to the perceptual processes underlying the illusory appearance. An expanded version of Gregory’s categorization of types of illusion is developed, and a range of classic and more recent illusions that illustrate the differences between these philosophical viewpoints is considered in detail. Such cases make it clear that the discrepancies from the measurable image structure cannot be simply regarded as idiosyncrasies of sensory coding, but are categorical exemplars of perceptual illusions. The widespread existence of such illusory percepts is indicative of the evolutionary limits of adaptive sensory coding.

## Introduction

Wundt (1898) defined geometric-optical illusions as “errors in the apprehension of spatial extents, directions, and differences of directions” (p. 55); Gillam (1998) has proposed that “the term illusion typically refers to a discrepancy between perceived reality and objective or physical reality”; Gregory (1997) has defined illusions as “discrepancies from truth”; and Rogers (2010) has raised issue of what illusions are, and has advanced the conclusion that they are situations in which the eye is led by the available information to misinterpret the physical situation implied by that information. However, he argues that sensory information is never complete, that the eye is always in the situation of interpreting the information available through the filters of the sensory apparatus, and hence that there are no visual illusions as such, only varying levels of information about the underlying reality. In particular, if the information from one visual scene is artfully arranged to appear to derive from a different kind of visual scene, Rogers argues that this is not an illusion because there is no source of information that the perceived scene has the form of the actual scene, and hence it is meaningless to define the distorted percept as an illusion.

## Physical Reality

Richard Gregory, the great champion of visual illusions in the current era, had this to say about defining illusions:

“It is extraordinarily hard to give a satisfactory definition of an “illusion.” It may be the departure from reality, or from truth; but how are these to be defined? As science’s accounts of reality get ever more different from appearances, to say that this separation is “illusion” would have the absurd consequence of implying that almost all perceptions are illusory. It seems better to limit “illusion” to systematic visual and other sensed discrepancies from simple measurements with rulers, photometers, clocks, and so on.”

(Gregory, 1997, p. 2).

Thus, the key distinction in the concept of illusions is between how things appear and how they “actually are.” But how they “actually are” is a multidimensional concept that can be addressed by all manner of sophisticated scientific and philosophical approaches that have little in common with the sensory input. It is like comparing not just apples and oranges but apples and quarks—they are utterly incommensurate entities. The whole concept of an “illusion” becomes itself illusory and meaningless when considered in terms of the underlying physical reality.

For example, one could say that color is an illusion because it is a way of encoding the infinite spectra of incident wavelengths into a 3-dimensional code that throws away most of the spectral information. The color, therefore, is a condensed distortion of the underlying physical reality of the spectral distribution of the incident light., as it is understood. But, as Rogers (2010) points out, this is just what we mean by a “sensory code,” not an “illusion.” All sensory input involves a transduction from some physical energy source into a neural code, with an inevitable loss of information to some degree. Do all these transformations qualify as illusions? In general, Rogers’ position seems the only reasonable one, that the answer is “no.” Nevertheless, in everyday parlance, color illusions are said to occur when an object that is usually experienced as one color is made to ***appear*** to have an ***objectively*** different color. That is, an illusion as generally conceived is **a *deviation from the normal operation of the sensory code*,** not the nature of the sensory code itself. For example, as we look around a woodland scene full of leaves, they continue to appear a similar green color under the various fixation conditions. If we engage in the abnormal behavior of staring at one leaf against a neutral background, then looking at a uniform patch of the field, we will see the momentary impression of a red leaf from the afterimage of the fixated leaf. It soon becomes clear that this was an illusory color, however, as it fades into the uniform background and we discover that it was contingent on the abnormal fixation conditions.

On the other hand, an abnormal color appearance is not an illusion if the different color is due to an identifiable cause, such as colored lighting. Then it is understood to look different than under the usual lighting conditions, but to remain its original color “in reality.” Thus there is a lot of “reality testing” involved in genuine illusions. In order to qualify as such, their appearance has to remain distorted even after we have verified the underlying reality to our best ability.

Perhaps a more cogent example that brings out the need for personal verifiability is the experience of solidity. Objects such as tables and coins are experienced as solid, but physicists may (and often do) claim that this is an illusory perception because their atomic studies reveal that the objects are in fact 99% or more “empty space.” Does this experience of solidity, then, qualify as an illusion? I would argue “no,” because this “empty space” is filled with force fields (or some equivalent description of the “action at a distance” that maintains the separation between atoms, between the nucleus and the electrons of atoms, and so on). The properties of these force fields are such as to repel the advance of our finger as we attempt to penetrate the spatial region of the object. Although the physicist can evoke a theoretical description of the underlying reality revealed by his or her experimental studies, the fact is that, at the level of description corresponding to our personal interaction with the entities, “solid” is the best specification of the essential nature of the repulsive force fields that govern the energetic interactions of our finger with the array of atoms corresponding to the specified objects. Thus, the experience of solidity is a functional property of the interactions between the force fields within the object and the force fields within our finger. This interaction is as real (and as far from illusory) as anything we experience in our lives.

## Defining “Illusion”

On the above reasoning, therefore, “illusions” are ***forms of interaction*** that are specific not to the known, or purported, underlying physical reality but to the level of human interaction with the physical reality. My proposal, therefore, is that an “illusion” is some sensory experience by humans (or other organisms) that is ***verifiable as aberrant*** by the experiencing organism. It is not simply that experts (such as physicists) can assert that the nature of the object is different from the experience it evokes. The experience is an illusion by virtue of the fact that its appearance can be verified personally by the experiencer in its sensory domain to be different from the underlying reality inferred from its generic appearance.

This definition excludes the cases where sensory coding gives a different answer from some other form of energy assessment. Thus, if an apple appears red under the normal variety of lighting conditions but yellow when viewed under a sodium lamp, we can say that that the sodium lamp gives an illusory view of the color ***because*** the apple can be moved among the different forms of illumination to ***verify*** that its appearance under the sodium illumination is aberrant. Or if rubbing a solid object in a certain way makes it feel soft, we call that an illusion ***because*** it feels hard under most validation conditions. Illusions, then, are special situations that make objects or images appear different from their typical appearance, ***as verified in the sensory domain*** under the majority of conditions. As such illusions are experienced in two ways. They are experienced as a real configuration in the first instance, before any verification activity, but they are experienced as an illusory distortion (or other form of configuration) after verifying that the generic reality differs from the immediate percept. In the first case, the observers are subject to the illusion but unaware of it, while in the second they become aware of the contradiction between the two modes of interpretation—real and illusory (this analysis follows Todorović, 2020, p. 1181).

**FIGURE 1:**
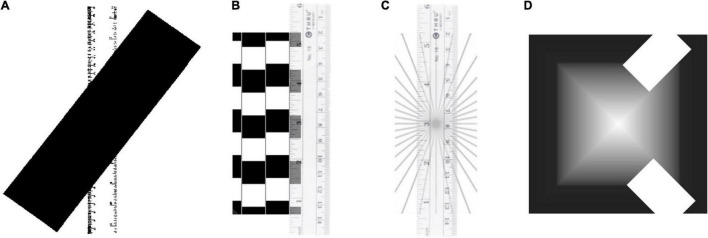
**(A)** Poggendorf illusion with a ruler as the test figure (from Blakemore, 1973). **(B)** Café Wall illusion (Gregory and Heard, 1979) with a ruler overlaid on one of the apparently slanted lines, verifying its physical straightness. **(C)** Hering curved line illusion with the same ruler as its test figure. **(D)** Pyramid illusion (Vasarely, 1966), a variation of the Mach Band illusion. The white rectangles depict how two business cards could be overlaid to verify that the luminance is uniform across the width of each triangular face of the pyramid.

The concept of direct sensory verifiability seems to be what Gregory was aiming at when he specified the “discrepancies from simple measurements with rulers, photometers, clocks, and so on.” These are all measurements that may be made by the observer of the illusion in its sensory domain in order to verify “true” situation from which the illusion is discrepant. A similar requirement was incorporated in the definition of illusions by Ebbinghaus (1913), Titchener (1928), Brunswik (1935), Blakemore (1973), and Rock (1983). Ebbinghaus (1913), for example, said: “when one observes simple plane figures, consisting mainly of only a few lines, often conspicuous differences are manifested between spatial relations as seen directly by the eye and as can be shown indirectly to be present by way of measuring aids. (pp. 51–52).” In comprehensive history of illusions and discussion of the concept itself, Wade (2005) points out that “an illusion requires a yardstick or reference relative to which it can be assessed.” and Blakemore (1973) says something similar in specifying that: “sensations can only be known to be illusory if there is a scale against which to judge the sensation and discover that it is false.” He illustrates this statement with a version of the Poggendorf illusion incorporating a ruler, whose role is to emphasize its known function as a straightedge disrupted by the intervening oblique bar (Figure 1A). However, as striking as the illusion is in this configuration, it does not illustrate the actual process of discovery, or verification, which requires removal of the oblique bar, or overlay of a physical straightedge, for direct verification of the straightness of the depicted ruler. What is needed is a measurement that may be made ***by the observer*** of the illusory distortion in order to verify the “true” situation from which the illusion is discrepant Note that the measurements are intended to be restricted to the domain of the phenomenology of the illusion.

Thus, appreciating an illusion is effectively a two-step or ***dual process*** of comparing the particular percept under one observational condition with a more intensive evaluation of the situation, giving rise to the percept under a wider range of observations and more rigorous measurements—the verification stage. It is important to stress that, under this new definition, the illusion is not just the optic (or other sensory) array that evokes the percept, but the whole physical situation in which it occurs, and that allows the observer to perform some sensory verification operation on the sensory array that allows its physical nature to be assessed. For example, a particular figure alone or in some context that induces a different (illusory) appearance is a powerful form of verification that the change in appearance is illusory.

Blakemore goes on to say “The whole of our perception is really false, for it does not copy reality but symbolize it. Only when the falsehood is manifest do we call it an illusion.” As argued above, this statement seems too strong for two reasons. One is that there are a variety of forms of neural encoding that are not captured by the term “symbolize,” which should be replaced by the more general “encoded” (since neural encoding does not involve explicit symbols of any kind). The other is that this variety of forms of encoding cannot be considered “false” because it is not their role to replicate the reality identically (which would merely be another piece of the same reality), but to ***encode*** it in a form that allows for further processing for use by the organism to deal with the reality it encounters for its own purposes. Thus, I would rephrase the statement: “The whole of our perception is a sensory transformation, for it does not copy reality but encode it. Only when an encoding aberration becomes manifest does the viewer call it an illusion.” If the observer does not engage in an appropriate verification procedure, then s/he is subject to the illusion, and may act on the wrong or distorted percept, even without knowing that this is the case.

How does this analysis square with the new definition of illusions as ***sensory experiences that are verifiable as aberrant by the experiencing organism***? If a verification procedure is available to the organism, the outcome is that the experience is either (a) true or (b) illusory (measurable as either veridical or distorted within the margin of error). The third case is that (c) no verification procedure is available, providing the middle ground of neutral cases where it is meaningless to pose the question. Specifying the definition of illusions in this active framework of verifiability, rather than the passive view of the information available at the sensorium, explicates the philosophical error of applying the Law of the Excluded Middle (or Aristotelian opposites). Rather than it necessarily being the case that all perception is either true or false (veridical or illusory), the verification concept exposes a meaningful middle ground of percepts that are indeterminate in this verification framework—neither real nor illusory, but incommensurate transformations of the incoming information. In this sense, many experiences are neither real nor illusory, but *sui generis*—“it is what it is,” as the saying goes—and cannot be forced into the verification framework of a binary choice between “true” or “false.”

Indeed, both Morgan (1996) and Rogers (2010) take the position that all (or most?) so-called “illusions” fall into this incommensurate category, that they are simply transformations of the physical information by the sensory apparatus, and that since all perception involves such transformations of one kind or another, none may be considered veridical and it is therefore invidious or unnecessary to single out a subclass of these transformations and call them “illusory.” Morgan cites as the prime example the variety of illusions of orientation, where the orientation of some element appears distorted relative to its physical orientation (see Figures 1B,C). For Morgan, the question is, what is the element that is distorted? Since the elements of the scene go through various forms of filtering before reaching consciousness, the element being judged is very different by the time it reaches consciousness, and hence its perception may be treated as veridical (or unspecifiable) in terms of the information available at that stage.

But this is surely putting the horse behind the cart! Everyone defines the illusion in terms of physical reality, not some neural reality at some unspecifiable location in the brain. The whole value of illusions is in the light they shed on aspects of the aberrant neural processing (relative to the typical cases where a verification procedure validates the initial perception). One cannot first use the existing illusory percept as justification for developing a model of the aberrant processing, then declare the illusion not to exist because we can now explain its neural basis! Had there been no illusion, there would have been no need to develop an explanation for it. This is a serious case of moving the goalposts of the argument from illusory perception of the external reality to non-illusory “perception” of a presumed state of neural information somewhere deep inside the perceptual process. The fact that we may be able to explain the apparent tilting of the lines in Figure 1B in terms of collector units (Morgan, 1996) does not straighten our percept of tilts. It merely moves them into the category of illusions that have actually led to enhanced knowledge of neural processing idiosyncrasies (as opposed to the many that remain contentious, such as the Poggendorf illusion of Figure 1A). And in any case to agree to Morgan’s dismissal requires acceptance that some particular form of explanation is sufficient to account for all aspects of the illusory situation, which has often been hard to come by in the illusion field.

The same point goes for the Mach Bands perceived in the Pyramid illusion (Figure 1D) discussed by Morgan (1996). The fact that neural filters at some level in the visual processing system can produce Mach Band effects does not mean that the illusory percept of the bands does not exist. The fact is that bright bands are seen along edges that are the same physical intensity throughout. The explanation may be simple, and may go back to Mach, but it is still a verifiably non-veridical percept of something that does not exist in the physical object.

**FIGURE 2:**
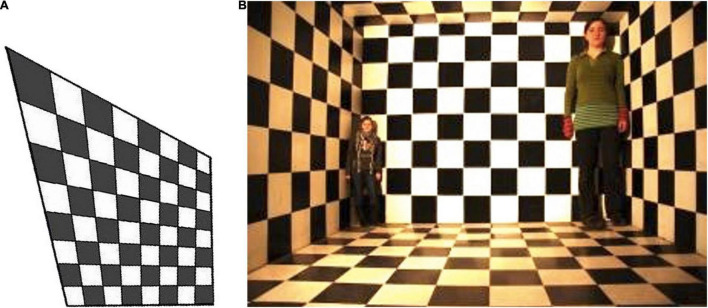
**(A)** The construction geometry of the Ames Room illusion. **(B)** A checkerboard version of the illusion, showing the apparent size difference between two people of equal height induced by the normalization assumption that the checks are physically rectangular. The illusion can be revealed by the operation of asking the two people to exchange places. Image credit: https://www.freeimages.com/photo/ames-room-illusion-1156662.

In light of this direct verifiability definition, let us evaluate the particular cases considered by Rogers (2010) to dispose of the concept of illusions. The Ames room (Figure 2) is a physically trapezoidal room that is artfully constructed so as to match the visual projection of a normal rectangular room when viewed from a particular viewpoint (and to be construed as rectangular on the basis of the tendency for perceived angles to default to right angles in the absence of strong information to the contrary). Indeed, physical Ames rooms are constructed with a peephole enforcing the intended monocular viewpoint, so that no other stereoscopic or motion information is available to cue the viewer as to the true geometry of the room. The distortion is revealed by placing two people of similar height in the far corners of the room, who then appear to have strikingly different heights.

Rogers is arguing that the visual information from the available viewing point is constrained so that the rectangular percept of a room with the different-sized people is the only possible interpretation of the scene, and therefore that is cannot be classified as illusory. However, his definition of illusion relies on the analysis of the **stimulus information alone**, which, as we have seen, may be artfully constructed to limit the perception to a distorted view of the actual situation. If we incorporate the concept of **sensory verifiability** into the definition of the illusion, the constraint is removed. Even from the designed viewing point, the illusion is revealed by our prior knowledge that the two people in the scene were of similar height. We can validate this assumption to verify that the percept of the room is illusory by arranging for the two people to walk together in the middle of the room to verify that their height is the same, and exchange their perceived heights as they proceed to take each other’s places in the opposite corners. Thus, the verifiability involves a **sensory verification procedure** that should be considered an essential part of the experience that makes some physical situation illusory.

Again, in terms of 3D information, he argues that, since any viewing system (human or artificial) has only the binocular disparity information to work with, it cannot know whether it is viewing a real scene or one artificially constructed to provide the same disparity cues. Hence there is no difference between the illusory stereopsis and the viewing of a real scene providing the same differential monocular information to the two eyes. While this logic has superficial plausibility, it falls down on the verifiability premise both within the viewing situation (as elaborated below) and given the larger conceptual frame of the viewer loading the images into the stereoscope (and therefore able to see that the object being viewed is in fact a pair of flat images).

**FIGURE 3:**
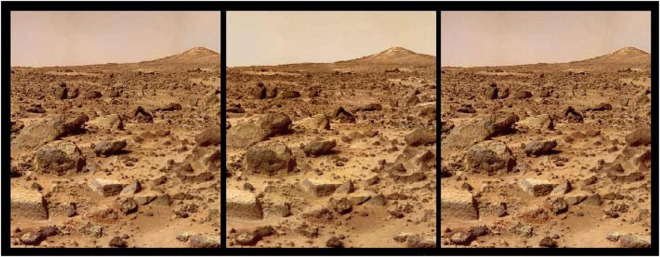
Stereogram image of the surface of Mars in a triple (R-L-R) format designed for easy free-fusion. Note that the monocular information before free-fusion makes it explicit that the image is a flat frontoparallel 2D surface, whereas the 2D texture information suggests a roughly uniform gradient in depth. The valid disparity pairing shows that the gradient is, in fact, strikingly non-uniform, with an early rise and a drop back down before rising again into the distance. The inverse pairing reveals a strong interaction between the 2D form information and the stereoscopic disparity information, in that the inverse depth image does not settle into a coherent structure. This variety of flat and depth impressions from the same pair of images clearly indicates that the perceived depth is illusory.

**FIGURE 4:**
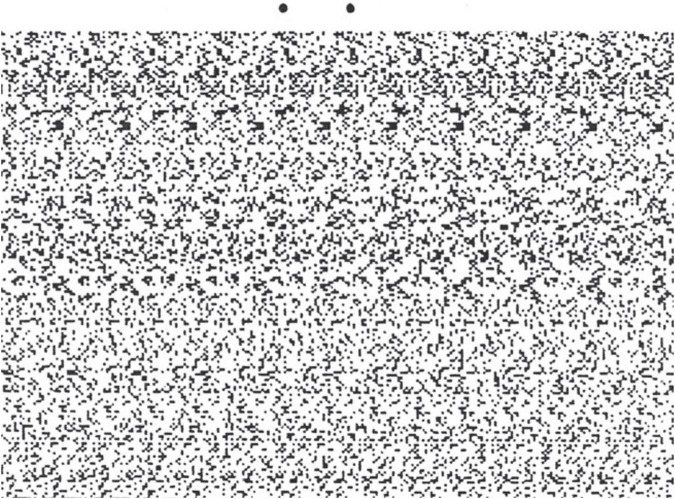
Autostereogram of horizontal corrugations. For free-viewing of the depth impression, converge or diverge the eyes so as to see the pair of dots at the top as a triplet, when the depth impression should emerge in the texture region. From Tyler and Clarke (1991).

1. As is well known in the field of depth processing, objects at different distances have different focus points relative to the retina, producing blur when they are not focused on the retina. This blur is not static, but varies with the vergence angle between the two eyes. If the eyes are allowed to move while viewing the stereoscopic image, therefore, the blur cue cannot be mimicked in a flat stereopair, and thus could provide an internal basis for verifying the 3D structure giving rise to the depth percept, revealing the stimulus to be illusory.
2. An even more compelling cue that the perceived depth is illusory is the relative motion that should be perceived if the head is allowed to move while viewing the display. Static objects should be seen to counter-rotate (strictly, counter-shear) as the head moves in one direction. The lack of such counter-shear is another cue to the illusory nature of the stereogram that could, in principle, be used to distinguish it from real depth. In fact, the expectation of this rotation from motion parallax is so strong that visual system generates the percept of the opposite counter-shear, in which the parts of the image with nearer disparity are perceived to move *with* rather than *against* the head movement (termed “induced stereomovement” by Tyler, 1974), as can be observed by moving the head while free-fusing Figure 3. Ironically, this paradoxical movement of the static image can actually enhance the depth impression relative to static viewing, because the presence of perceived relative image movement is treated as perceptual evidence that the image is not a flat 2D field, even though the movement is in the “wrong” direction. However, if the objects have 3D structure rather than being simply 2D planes, they appear to be distorting by 3D shear rather than being rigid, which is a clear perceptual cue that the view is not of a normal rigid 3D world.

3. Many forms of stereoscope are constructed to allow the flat cards of a stereopair to be inserted for viewing, providing clear information before insertion to verify that the view is indeed an illusion.
4. A further feature of stereoscopes is that the artificial disparity is provided in the horizontal direction of the disparity information. When the images are rotated by 90^°^, this is converted to vertical disparities, which are not processable locally for perceived depth, and hence the illusory nature of the depth information becomes evident (unlike the 3D world, in which disparity is always generated by the separation of the eyes, and is therefore always appropriately “horizontal” in head coordinates).
5. Another type of stereoscopic presentation device that allows verification of the illusory status of the perceived depth is the autostereogram, or single-image 3D display that provides for depth perception from binocular disparity when the eyes are reconverged at the designated distance away from the plane of the image (Tyler, 1983; Tyler and Clarke, 1991). A simplified form of free-fusion is the triple stereogram exemplified in Figure 4, allowing for free fusion across adjacent pairs of images to provide both crossed and uncrossed fusion. For those able to perform this trick, not only can they switch the visual impression from 2D to 3D by appropriate reconvergence, they can also generate the paradoxical induced stereomovement of the previous paragraph when in the 3D mode and collapse it by head rotation, and they can additionally verify the 3D structure of the image by tactile inspection. Under our verifiability definition, the availability of these multiple forms of verification fully qualifies the perceived depth as illusory rather than real.

Rogers’ third example is the sense of color, of which he develops the argument that “since all perceived colors have metamers, all color perception would have to be regarded as illusory., as Charles Wheatstone pointed out a hundred and fifty years ago.” This inference is based on the idea that metamers, or color mixtures that match a simple color or other color mixture, imply that colors are illusory. But this is analogous to saying the vector sum of two forces is illusory because the same sum could have been derived from a different set of forces. These are just the laws of force, without prejudice as to the reality of their existence. The same should go for color: the existence of metamers is just the way the color sense operates, without any implication for its relation to some underlying reality. Thus Rogers concludes that it is more sensible to treat colors as a sensory code to which the concept of illusions does not apply.

Thus, our experience of color is a coding system that does not match the structure of the linear frequency scale of light and the infinite variety of combinations of frequencies. In this sense, all colors are non-veridical, but this is not a sense that is accessible to us on a personal level, since we have no other way to sense the gigaHertz light frequency (and it seems a biological miracle that we have developed some way of doing it at all!). What makes more practical sense is to contrast the habitual way of seeing colors with aberrant cases, such as the aftereffect of staring at vivid colors generating afterimages of their complementary colors (based on the structure of our color encoding) on a surface that is verifiably white without such a manipulation. Thus, color *per se* is an encoding sense, neither “real” nor “illusory,” but color afterimages, spatial color induction, temporal color induction, and so on, are thus indeed classifiable as illusory deviations from the generic impression of particular spectral distribution, and hence as color illusions.

However, in relation to the neural mechanisms underlying perception, Rogers (2010) follows Morgan (1996) in treating illusions as misperceptions whose mechanistic basis remains obscure. Once the neural mechanism is known; “The interdependence of information and mechanism leads to the conclusion that, if we fully understand how our perceptual systems work, there would be no illusions—there would only be descriptions of the characteristics and limitations of our particular perceptual mechanisms.” (p. 288). Conversely, the present viewpoint is that, although the mechanism of the illusion may have been resolved, the percept is still illusory. The stick remains optically bent in water, however, well we have understood Snell’s Law of Refraction. The solid rock beside the waterfall still appears to move despite our knowledge of the synaptic signal decay in the motion-selective neurons in the cortex. These remain perceptual illusions even though they are no longer conceptual conundra.

Finally, consider Todorovic’s strategy of providing a verification procedure in the form of 2 × 2 (Aristotelian) contrasts for spatial illusions, where the context is inducing or non-inducing and the test targets are equal or unequal. This logical cross-pairing gives viewers the critical opportunity to compare the percept with and without the inducing feature of the context, if they trust the source to have been printed correctly. But given the vagaries of the various media with which they may be viewing the images (such as automatic gain control in tablet computers, or the printed false amplification of the illusionary effect discovered by Favreau (1977), which she termed the “Müller-Liar illusion”), it is no substitute for a direct verification procedure in which they can physically manipulate a context to check the percept directly themselves.

## Grand Categorization of Illusions

Gregory (1997) brought some order to the field of illusions by developing the categorization of illusions reproduced in Table 1 that recognized four categories of illusions: ambiguity, distortion, paradox, and fiction. Although there have been other approaches to the categorization of illusions, as reviewed in Todorović (2020), for example, Gregory’s is by far the most comprehensive. In each case, we need to consider why this category is considered to be an “illusion.”

**TABLE 1:** Gregorian categories of illusion.

Kinds/ levels	Ambiguity	Distortion	Paradox	Fiction
**Optics**	Mist	Star twinkle	Looking-glass	Rainbow
**Signals**	Retinal rivalry	Café wall	Rotating spiral	Afterimages
**Rules**	Kinetic depth effect	Müller-Lyer	Penrose triangle	Kanizsa triangle
**Objects**	Duck/rabbit	Size-weight	Magritte mirror	Faces in the fire

Gregory’s illusion categorization was bivariate: types of illusion vs. the level of processing involved (what Gregory termed “kinds of causes”). The levels of processing he considered were “optics, signals, rules, objects,” together with the language level of illusion that he introduced in an earlier table which may be recast as physical, sensory, perceptual, cognitive, and linguistic levels of processing (where “cognitive” is here limited to high-level pre-linguistic processing).

Gregory’s examples are almost all from the visual modality, but we must recognize the dimension of the other senses: hearing, touch, taste, and smell, each of which are subject to their own array of illusions that could fill the same 4 × 4 table for each sense. To these sensory domains need to be added the more abstract domains of time perception, self-perception, social perception, etc. Within each sense modality, there is a further subdivision of perceptual domains, such as intensity, quality, location, depth, dynamics, and the spatial contrasts between them (In the visual modality, these correspond to brightness, color, position, binocular disparity, motion; luminance contrast, chromatic contrast, orientation, relative motion. The domains may not all be definable in each sense modality, but many are). Thus, the categorization of illusions extends to at least four dimensions: type, processing level, modality, and domain. For example, ***motion*** is one perceptual domain within the visual modality, subject to illusions of optics, signals, rules, object structure, and even linguistic descriptions. At each of these levels, one can recognize motion ambiguities, motion distortion, motion paradoxes, and fictional motions. Thus, any one domain of any one sense modality is subject to the full two-dimensional categorization of the Gregorian scheme.

**FIGURE 5:**
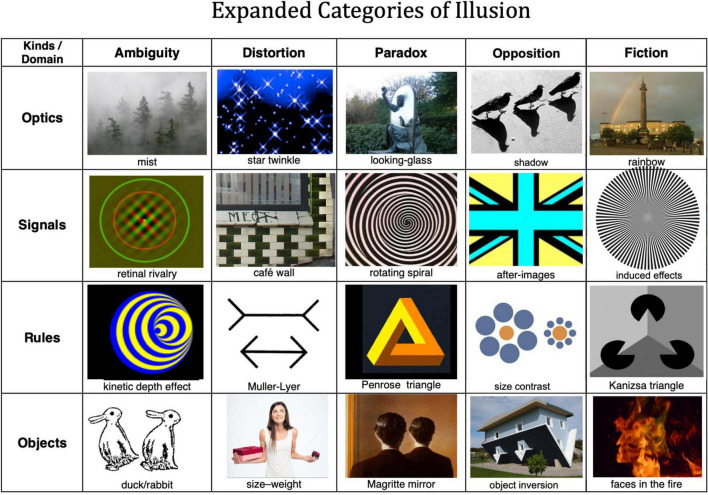
Expanded table of illusion categories, with an illustrative example of each one, respectively of the categories of ambiguity, distortion, paradox, opposition, and fiction. Row 1—optical illusions: trees in mist, twinkling stars, Alice through the looking-glass in a Lewis Carroll memorial, shadows, rainbow in Liverpool. Row 2—signal illusions: anaglyph stimulus for binocular rivalry, the original café wall in Bristol, evocation of a motion aftereffect, flag image to evoke standard-color afterimage, induced scintillations. Row 3—rule illusions: stimulus to evoke kinetic depth when rotated, Müller-Lyer arrows, Penrose impossible triangle, Ebbinghaus size contrast illusion, ambiguous shading-enhanced version of the Kanizsa illusory triangle. Row 4—object illusions: double ambiguous duck-rabbit, depiction of the size-weight illusion, Magritte painting of a non-reversing mirror, physically inverted house, faces-in-the-flame image.

Consideration of Gregory’s table suggests that he missed an important kind of illusion. The “Fiction” category includes some examples that are pure fiction emerging with no relationship to the rest of the scene, but others that arise in direct opposition to some aspect of the inducing image. This suggests the need to include “Opposition” as a separate category from the non-oppositional forms of fiction, leading to the expanded form of illusions table provided in Figure 5, which has been enhanced by thumbnail images of each example.

### Illusions of Ambiguity

Ambiguities are illusions because the role of the senses is to provide a specification of the physical reality around us, and the presence of ambiguity implies that we have two or more competing specifications, preventing us from establishing what the surroundings are “really” like. Mist makes objects difficult to distinguish. Retinal rivalry is the perceptual alternation that arises when two non-fusible images are presented to the two retinas. The kinetic depth effect (Wallach and O’Connell, 1953) is one example of an ambiguous percept in the domain of perceptual rules. The rule is that the 2D motion on the retina is interpreted as deriving from the simplest form of 3D motion of some rigid object (where “simplest” is derived from experiential priors of our interaction with the 3D world). In many cases, however, the perceived depth depends on the assumption of how fast the scene is moving relative to us (or vice versa), and hence the depth scaling of the precept is ambiguous (in the sense of indeterminate). These are verifiable illusions because the substrate is verifiably single, so the multiple state must therefore be illusory.

### Illusions of Distortion

Distortion is the classical case of an illusion in which a stimulus is perceived to deviate from the physical configuration with which it is constructed, such as steady lights seen as twinkling, straight lines seen as bent, equal lines seen of different lengths, equal weights of different sizes felt as different, and so on. The physical configuration can be verified by viewing the illusion from a different viewpoint, examining the contents of the weight containers, and so on.

### Illusions of Paradox

Illusions of paradox have something in common with those of ambiguity, in that two or more incompatible percepts are counterposed such that not all may be seen as an integrated scene. The difference is that in the case of ambiguity, only one percept may be seen at a time, whereas in the case of paradox both are seen simultaneously but form incompatible cognitive interpretations, such as a looking-glass seen as simultaneously solid and transparent, a field seen simultaneously static and moving, a structure seen simultaneously as protruding and receding, a mirror seen as simultaneously reflecting and non-object-reversing, and so on.

### Illusions of Opposition

Another form of duality in the illusion domain is illusions that arise in direct opposition to some aspect of the prevailing stimulus. From optics, an example is shadows that arise as quasi-objects in opposition to objects that are producing the boundaries by the projection of light-rays past them; from signals, an example is the chromatic afterimage the develops in opposition to prolonged viewing of a structured image; from rules, an example is the opposing effects of context size on the perception of the size of features embedded in that context; and from objects, an example is the inversion of objects in opposition to their familiar orientation in a gravitational field.

### Illusions of Fiction

These are simply cases where something is seen that is not physically present in the sensory information impinging on the organism, yet is sensed as though it were present in the world, such as: rainbows, uniform fields seen as having colored patches, borderless triangles popping out in depth, faces appearing in the flames of a fire, and so on. These cases of the absence of the perceived object or structure, as are verifiable by information from another sense.

## The Natural Image Approach to Illusion Definition

One of the key frameworks for conceptualizing illusory perception is the natural image framework expressed by Purves et al. (2008) as follows:

“The hypothesis is that all visual percepts are generated empirically to facilitate successful behavior, and were never intended to correspond to the physical properties of the world or our measurements of these properties. From this perspective, illusions do not reflect any inadequacy or imperfection of visual function, but are rather signatures of its core strategy. The experimental approach to validating this hypothesis is to use natural image databases as proxies for accumulated human experience with some aspect of the visual world.”

This conceptualization is an appealingly adaptive hypothesis inspired by evolutionary theory, but it has several key self-contradictions as expressed, and fails to lead to its empirical conclusion.

(i) One contradiction is the implied dissociation between successful behavior and physical properties of the world. It would seem self-evident that successful behavior depends on an optimal understanding of the physical properties of the world, to the extent that they constrain our behavior. Thus, if you are running through a dense forest, it is critical to have an accurate understanding of the physical placement of solid obstacles in your path in order to avoid severe injury.

(ii) A second contradiction is the idea that visual percepts are generated “empirically,” which usually means by behavioral trial and error. In some sense all life is empirical, but if the authors mean to enfold the concepts of evolutionary adaptation, genetic encoding, epigenetics, ontogenesis, maturation, behavioral adaptation, and cognitive processing into the term “empirically,” they should be more explicit.

(iii) A third issue is the use of the term “never intended,” which (apart from its “intelligent design” implications) appears to be a value judgment about the relevance of physical reality to perception. In general, perception needs to provide an accurate representation of physical reality in order to be successful in the aspects it represents, and is thus always “intended” to be accurate. There may be many aspects that it does not represent (such as gamma-ray energy), but these would not generally be termed “illusions.”

(iv) A further problem is the statement that natural images are an adequate proxy for the physical reality that we deal with. As hinted in the first item, the physical world is largely composed of discrete three-dimensional objects that retain stable geometrical configurations as we move around in relation to them. The *images* of objects, on the other hand, have highly variable concatenations of object features, often jumbled up among different objects or generated by spatial occlusions among different objects. The structure of images thus bears only a very partial relationship to the physical three-dimensional structure of the objects that we need to manipulate in our interactions with physical reality. Moreover, the image structure is subject to profound perspective distortions, convergence and foreshortening, when mapped to the two-dimensional image space, which have nothing to do with their three-dimensional configuration that are the basis for these interactions.

Another form of self-contradiction implied in the quoted statement is in the view that natural image *statistics* are the appropriate form of analysis from which to derive the regularities underlying visual illusions. To perceive the world in terms of image statistics would thereby generate massively illusory percepts of jumbles of image features. It is not until we move to the analysis of natural *object* statistics that a worthwhile mapping will be achieved. The term “statistics” is not employed directly in the quote, but are implied by the reference to “natural image databases.”

**FIGURE 6:**
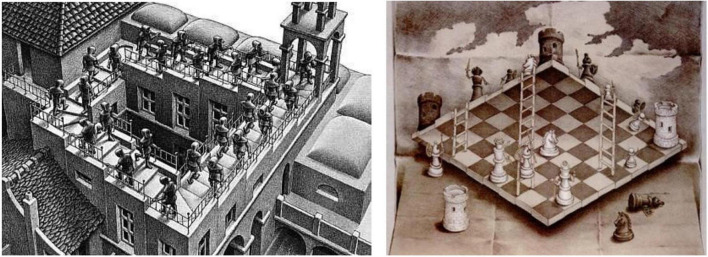
Two examples of ambiguity in the 3D interpretation of complex images: *Ascending Staircase* by Mauritz Escher, and *Two-Sided Chessboard* by Sandro del Prete.

As in the Disraelian aspersion (that there are “lies, damn lies, and statistics!”; see Twain, 1904/2010), natural image statistics are often a poor proxy for the complex configural relationships among object features, which may extend to higher-order statistical relationships that are computationally inaccessible. Even low orders of statistics may be deceptive, as in the case of the statistician who drowned in a river that had an average depth of one inch! Imagine, then, the problem of defining the logic of the Escher ascending staircase illusion (which does not become inconsistent until the images of 20 or so steps are connected) on the grounds of natural image statistics alone (see Figure 6).

(v) The final inadequacy of the natural image approach is its broad focus on *background* statistics. In general, these are the statistics of the features that we need to ignore (say the greenery of the foliage) as we forage for items of nutritional or other utilitarian value (say, fruits, and berries). These items of nutritional value are statistically quite rare, and are also confused with other items of similar rarity but no utilitarian value (such as, say, flowers, and display insects), which have other adaptive bases. The ability to pick off the particular statistics of utilitarian value to the perceiving organism is a further issue in the field of natural image statistics that has rarely been addressed by the statistically minded research community.

The net conclusion from this analysis of the natural image framework to illusion analysis is that, although it may be computationally tractable to some depth of analysis, it has numerous shortcomings as a meaningful structure for addressing more than a small subregion of the four-dimensional space of image categorization recognized here on the basis of Gregory’s initial conceptual scheme (see text discussing Figure 5).

## Optical Illusions

The term “optical illusions” is often used interchangeably with “visual illusions,” but here we will restrict them to aberrant effects due to the optics of the light mediating perception, and those in the neural visual processing, respectively. Thus, optical illusions will be considered those in which the interpretation of the world is distorted or aberrant due to some unusual physical property of the light mediating vision, whereas visual illusions would be those deriving from neural signals in the retina and the brain. Entoptic phenomena are an intermediate case that may be subdivided according to whether they derive from optical effects within the eye (corneal light streaks, macular pigment, Haidinger’s “brushes” seen in polarized light, and so on) vs. neurally mediated effects (such as luminance and chromatic afterimages of viewing high-contrast images, eigengrau in the dark, the Fiery Rings of Purkinje from optic nerve traction when making large saccades in the dark, and so on).

A key optical illusion that has been the subject of discussion (Morgan, 1996) is the bent stick illusion, whereby a stick protruding through the surface of water appears bent by the different index of refraction in the air and the water. A related effect is the appearance of mirages of pools of water over hot regions such as hot desert sand and distant roads in hot climates due the differential refractive index gradient of the heated air close to the surface. In a neural sense, these are merely aberrations in the optic array impinging on the retina, and therefore have been claimed not to be illusions at all. Under Gregory’s categorization, however, they are ***optical*** illusions, in the sense that they are incorrect interpretations of the physical reality underlying the optical information. Under the present scheme, they are indeed illusions because it is directly verifiable that the physical reality is otherwise than it appears. The stick can be withdrawn from the water to verify that it is, in fact, straight, and a trek toward the site of the distant “oasis” verifies that it disappears into dry sand. The verification concept thus reveals that the perceptual interpretation of the physical reality was incorrect, and hence illusory.

## Gestalt Field Illusions

**FIGURE 7:**
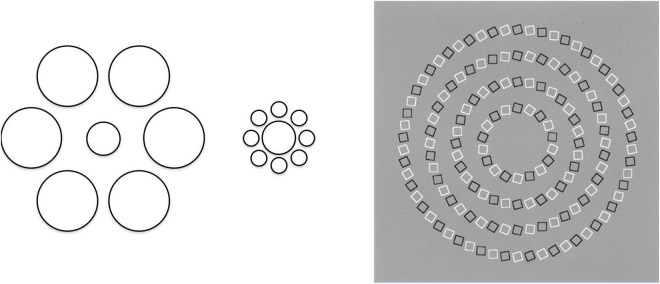
The Titchener and the Pinna illusions. In the Titchener, the central circles as the same size; in the Pinna, the rings are all concentric circles; illusions of distortion of visual signals.

The classic 19th century illusions were typically illusions of size and orientation, such as the Poggendorf and Hering illusions illustrated in Figure 1, or the Titchener and Pinna illusions of Figure 7. On the one hand, though usually cast in the form of local interactions, such constructions can be seen as various forms of Gestalt field illusions in which the interpretation of a local figure is influenced by the forms in the surrounding field. In a sense, the field of influence is a form of visual relativity in which the form of everything local is assessed relative to its the information in its surroundings. Most readers may well agree that this is a form of platitude that is now generally understood, but it has profound philosophical implications in relation to the Bayesian adaptive viewpoint, since it implies that we have not evolved to see the featural properties of things as they are—stable entities independent of other things—but by making the best of it with a perceptual system that is at the mercy of relativistically adaptable features that have no stable basis. In a technical analogy, the perceptual system is AC rather than DC coupled, with no defined zero to any of is fundamental dimensions of interpretation [position, luminance, contrast, color (hue and saturation), orientation, size, motion, disparity]. All these basic qualities are relative in space and time, subject to large contrast effects of both the simultaneous and successive kind, and in many cases capable of complete adaptation such that the strongest stimulation can evaporate to zero percept, which is the hallmark of an AC coupled system.

## Assessment of the Bayesian Framework

With these basic facts in mind, we can evaluate the view of the perceptual system as governed by Bayesian priors. The use of the term “Bayesian prior” is usually used in a rather loose sense, with the understanding that it can be difficult to determine the full swath of prior perceptual experience that has led to a relevant prior for a given perceptual task. But it should be considered that the priors can be categorized in three forms—phylogenetic priors, ontogenetic priors, and short-term adaptive priors. **Phylogenetic priors** are those that are built into the genetic inheritance of the organism, such as the ability to transduce sensory information and map its distribution of specialized energy into the visual cortex. **Ontogenetic priors** are incorporated lifetime experience of the organism from an early age, such as the ability to recognize objects and to read culture-specific text. **Short-term adaptive priors** are the immediately preceding sensory information over a timescale much shorter than a lifetime. Whether the timescale is months, hours or milliseconds seems to depend on the particular perceptual task, but it is clear from the above review of the temporally AC- coupled nature of perception that the immediate priors can have a profound influence on perception. Moreover, such a tripartite categorization is only a convenient simplification, since there is obviously a continuum of time scales to consider, from deep evolutionary (such as the make-up of the phototransduction cascade) to the shortest interactions between adjacent spikes. Moreover, the longer time scales can and do adaptively influence the use of the shorter ones.

The Bayesian framework implies that all three levels of Bayesian prior should be adaptive, in the sense that they should have been optimized to help the organism organize the perceptual input to implement its operational goals. In many respects, it is hard to see how the profoundly relativistic nature of perceptual achieves this optimization. The cognitive system is arranged on a strongly memorial basis, such that important memories (i.e., significant Bayesian priors) can last a lifetime, explicitly after the first few years, and implicitly perhaps from even before birth. The perceptual system, on the other hand, seems to adapt strongly on a timescale of seconds to minutes. Fully structured stabilized images disappear in a minute or two (Riggs and Ratliff, 1952) and profound face adaptation has recently been shown to take place in only a fraction of a second (Webster and Maclin, 1999). It is hard to conceptualize how these behaviors can be viewed as reflecting meaningful Bayesian priors. They seem to have much more of the flavor of a perceptual system reflecting the limitations of its enforced cellular substrate, in the vein of the “bag of tricks” interpretation of perceptual processing (Ramachandran, 1990; Billock, 2003). Admittedly, the system needs to wipe out the information across the retina from each fixation before proceeding to the next, but a sample-and-hold neural strategy (i.e., DC coupled with saccadic resets) would be far more adaptive than one of a gradual exponential decay, and sample-and-hold must be readily implementable in neural circuitry based on what we know of the neural capabilities outside the sensory systems. So the whole AC coupling strategy by which the sensory systems operate appears notably puzzling from the evolutionary adaptive viewpoint.

## Inexplicable Illusions (Bayesian Paradoxes)

We may now consider a series of illusions that appear to contradict the basic assumption that perception has evolved to optimize our interaction with the world. While this optimal concept of evolution is obviously true to a rough approximation, it clearly fails in many detailed respects. After all, we manage to operate at home, in the street and at the office without destroying ourselves or our surroundings, for the most part.

### Perspective Convergence

**FIGURE 8:**
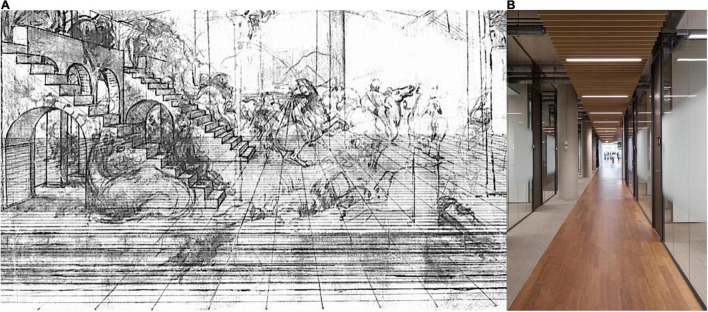
**(A)** The first extant perspective diagram (from Leonardo da Vinci, ∼1,475) illustrating the principle of the vanishing point. Note how the ground plane appears to slant upward rather than extending purely horizontally. **(B)** A vivid depth percept from a parallel corridor, a 3D form of a distortion illusion.

As considered above, we are all used to the idea that perspective lines should converge to a point in the two-dimensional image projection, as in Leonardo da Vinci’s diagram (Figure 8A). However, this percept of 2D images is quite maladaptive as a representation of the reality that we are traveling through. When we look along a corridor that we are just about to enter, the relevant reality is that the floor is flat and that the walls and ceiling will stay parallel and far enough apart for us to reach the end unscathed. All adaptive considerations would dictate that our perception should convey these relevant Euclidean realities. If we ask how the physical corridor is actually perceived, viewers report that even the physical parallels are perceived to be converging to some extent, with the floor rising, the ceiling sloping down and the walls closing in. The reader is encouraged to try it in person by looking down a physical corridor such as the one depicted in Figure 8B. This is not a question of distant mountains or diverging sun rays—it is the immediate reality of our local surroundings. Yet it seems that perception delivers an illusory interpretation that we have to work around, or ignore, in order to negotiate a simple corridor. As with many aspects of visual processing, the percept seems to deliver a compromise between the retinal configuration (converging lines) and the requisite reality (parallel lines). In the examples shown in Figure 8, this compromise results in the impression that the floor is sloping upward to the rear, as opposed to appearing horizontal in space.

In perceptual studies, this compromise is known as “phenomenal regression to the real object” (Thouless, 1931), implying that the perceiver fails to compensate for the (cognitive) reality implied by the shape of the object as a perspective projection when attempting to specify its retinal projection. This task, however, is not what evolutionary adaption has typically prepared the visual system for, which is the effective interaction with three-dimensional objects. In this sense, the illusion is the phenomenal regression to the retinal image. The perceived convergence is the retinal configuration intruding on the relevant real-world interpretation of the incoming information, as had recently by quantified in a landmark study by Erkelens (2015). From an adaptive perspective, the retinal image is just a waystation on the path to this relevant interpretation, and the perceptual scientist asking for the interpretation of a drawing is asking the “wrong” question. The net result is that there is a strong illusion of perspective convergence even when viewing nearby depth scenes even under full-cue, unrestricted interactive conditions, which seem to be those for which perception should be best adapted. This illusory distortion may derive from our modest experience with assessing far distances quantitatively, as we rarely have the task of filling an extended distance with equal-sized elements, as might be the task of a parking attendant at a large car park. One might expect those with experience of such tasks to have more accurate distance perception.

### Impossible Object Perception

**FIGURE 9:**
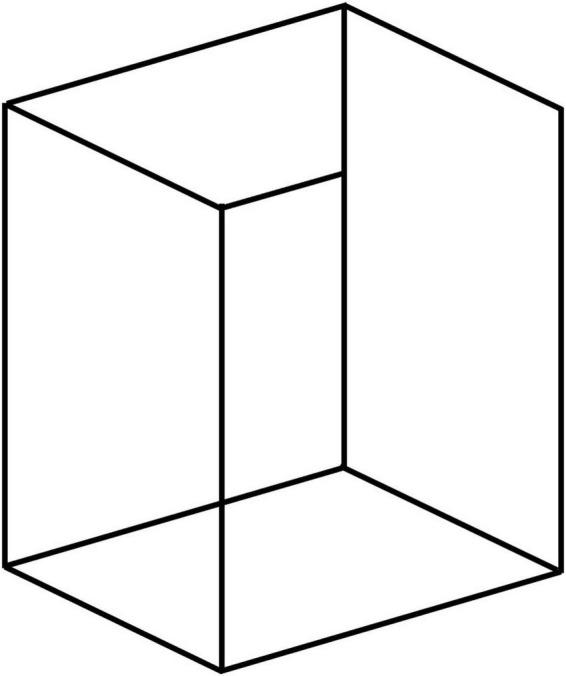
Impossible object, from Hochberg (1981). The dominant interpretation is of an *impossible* configuration with an unfinished bar, even though a *possible* interpretation of a bottomless cube viewed from underneath is readily available; an illusion of ambiguity and paradox of visual rules.

A strong example of an illusory violation of the adaptive realities is the ability to see an impossible object, such as the one from Hochberg (1970) illustrated in Figure 9. Since the object is impossible, it must by definition have an adaptive prior of zero likelihood of occurrence in the world. If we see it at all, we are violating this prior. The implication is that the visual system has pulled out a trick from its bag that works most of the time to approximate the prior, but fails in the specific instance.

Hochberg’s “impossible” object has the dominant interpretation of a Necker cube with one incomplete bar, or perhaps with a bar oddly connecting the front corner to the rear edge. These are obviously interpretations that have never been encountered in life (although perhaps not strictly impossible), and therefore have a vanishingly small probability of occurrence. On the other hand, its interpretation as a bottomless cube viewed from underneath, which will appear after staring at the image for a few seconds, is complete and readily possible as a physical 3D structure. Yet few people see this as the first interpretation, and, when they see it, most switch back and forth repeatedly between the two interpretations. Thus, even when the visual system has established that there are two dominant interpretations, one possible and the other impossible, it ***still continues*** to switch back and forth between them with similar dwell times. This perceptual behavior clearly violates the dictates of the prior probabilities preferring the possible to the impossible interpretation. Instead, the partial prior of the corner-front cube dominates the interpretation, overlaid with an implausible fix for the impossible loose bar. This behavior seems to imply that the visual system uses the trick of looking for front corners and reconstructing the remainder of the shape from there, despite the presence of a fully consistent interpretation in a less familiar pose. This heuristic employed by the perceptual system may be evolutionarily plausible as a perceptual strategy, but it should be emphasized that it does not fit the Bayesian formalism of selecting the best-fitting prior.

We are left with the picture of a three-way tug-of-war between the “bag of tricks” strategy, which is basically bottom-up, the Bayesian scheme of optimization according to the prior probabilities from past experience, and a top-down interpretive framework such as the perceptual hypotheses of Gregory (1980). This trio of network interactions needs to be integrated into a scheme that views all subjective contours, amodal completion, monocular depth effects, and so on as manifestations of an active, top-down process of 3D reconstruction. On this view, it is not until the image is parsed in terms of potential 3D objects that the object interpretation is formed.

An important source of illusions is such operations of midlevel vision in interpreting the structure of the incoming visual signals in preparation for the analysis in terms of object structure. In selecting the present set of illusions discussed in the following, I have tried to focus on those that illustrate some of the less common principles of visual processing that have been discussed.

## Induced Hallucinations

### The MacKay Chrysanthemum Effect

**FIGURE 10:**
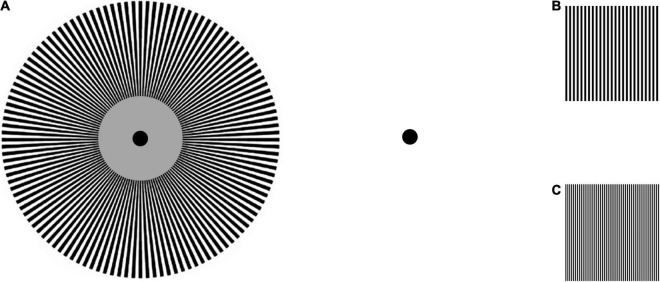
**(A)** MacKay ray figure, eliciting dancing “chrysanthemum” induction contours approximately orthogonal to the radial bars in the stimulus. Fixate the center of the ray figure for 10 s while rocking the head slightly around the visual axis, then fixate the black dot. A dynamic sheen of circular arcs like a chrysanthemum will be seen circling around the dot. **(B,C)** Linear gratings illustrating that the angle of the induced rays varies with the spatial frequency of the inducing grating.

A further form of illusion that challenges interpretation through Bayesian priors is the class of fictional signals induced in various ways by high-contrast grating images. A primary example is the MacKay Ray Illusion, which is both the simultaneous and the successive percept of a scintillating “chrysanthemum” form on prolonged viewing of the MacKay radial ray figure (Figure 10A). It is hard to see any adaptive utility of such an illusion, clouding our veridical percept of the high-contrast rays with an unstable overlay. The scintillating contours are often characterized as orthogonal to the physical rays, but the use of the term “chrysanthemum” is intended to emphasize that they are in fact at the oblique angle of about ± 60° to the rays, taking a roughly spiral form as a result of the linear increase in the ray spacing with distance from the center (Note that this perceptual effect should not be confused with the striking moiré fringes that are seen when the eyes move around this figure, as a result of the interference between the current view and the retinal afterimage of the previous view of the figure).

In fact, the angle of the induced contours varies with the spacing of the rays, as can be demonstrated with the linear arrays in Figures 10B,C, such that the angle of the rays from horizontal becomes steeper from about 30° to about 45° as the spacing between the bars is decreased. The rays become much more evident when the grating is flickered in counterphase, which may be approximated for the printed image by jiggling the page horizontally. This demonstration (Tyler, 1982; see Bressloff et al., 2001) indicates that the rays are acting as a kind of moiré fringe relative to some fixed structure in the visual system (presumably in the cortex, since it is an orientation-specific illusion). Projection through the cortical mapping function (Holmes G., 1918; Holmes G. H., 1918; Daniel and Whitteridge, 1961; Schira et al., 2010) indicates that the spacing of the internal structure, as implied by the ray spacing that produces a 45^°^ “chrysanthemum,” is about 1 mm on the primary visual cortex, i.e., roughly the spacing of the hypercolumnar structure (Hubel et al., 1977). (Nevertheless, it should not be thought that this identity implies that the moiré effect is due to interaction with the ocular dominance stripes *per se*, since the orientation of the illusory lines is an orientation specific induction that covaries with the orientation of the inducing ray pattern; Tyler, 1982).

### The Leviant Traffic Illusion

**FIGURE 11:**
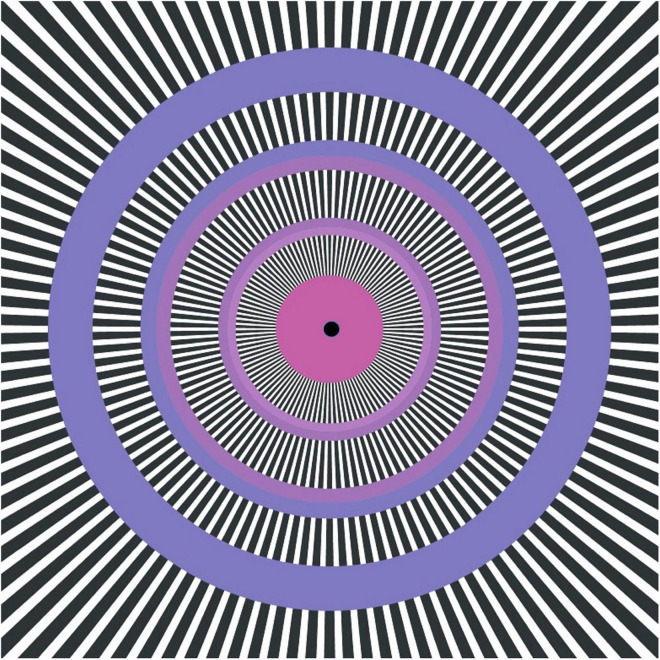
The Leviant “Enigma” figure, illustrating the “traffic illusion” and “perceptual tachyons” (see text); an illusion of fiction and ambiguity of the visual signals.

A motion illusion that is related to the MacKay chrysanthemum is the Leviant Traffic illusion (Figure 11), in which gray circular inserts into the MacKay ray pattern are seen to have apparent motion of some kind of illusory specks simultaneously in both directions around the gray rings (Leviant, 1996). It is important for the rings to be an isoluminant gray matching the mean luminance of the black and white rays—increasing or decreasing their luminance reduces the motion percept. In a sense, the gray ring is capturing the dynamics of the Mackay ray scintillations and making clear their motion component (Hamburger, 2007). Notice that the perceived motion is multistable, and can appear as a global rotation either clockwise or counterclockwise as well as local bidirectional motion.

### Perceptual Tachyons

A further twist on the Leviant illusion is an effect that I have demonstrated informally over the years but have not previously published, that may be called “perceptual tachyons.” Tachyons are notional particles arising from the negative sign interpretation of the Lorentz equations of theoretical physics, which have the mathematical property of always traveling faster than the speed of light (and requiring infinite energy to slow them down to that canonical speed). Motion aftereffects have the general property that they are always slower than the motion that induced them, becoming progressively closer to the adapting speed as the duration of the inducing motion is increased (analogous to the behavior of relativistic particles). Without pursuing the metaphor too closely, the property of a perceptual tachyon, therefore, is that it is a motion aftereffect that is always faster the speed of the inducing motion.

The perceptual tachyon effect is induced by fixating the center of the Leviant pattern in Figure 11 and rotating the image smoothly around the line of sight. During the rotation, you will notice that the rotational motion captures the rapid motion in the rings, such that it slows down to move at the same rate as the image rotation. The next requirement is to abruptly stop the image rotation ***while maintaining fixation***, and observe the effect on the motion in the rings, which will be seen to shoot off in the opposite direction to the rotation, but now at a much higher angular velocity than the rotation. This is the *perceptual tachyon* effect, since the slow inducing motion of the image rotation has generated a much faster aftereffect (presumably by selecting from one of the two directions that can be perceived in the Leviant motion effect). It is not clear either (a) how the retinal motion of the ray ends captures the Leviant motion to slow it down, or (b) how the release of this capture could influence the much more rapid ambiguous motion to select one direction so strongly, but these seem to be low-level interactions between the different sources of motion without any basis in Bayesian priors for object constancies derived from observations of the physical world.

### Induced Twinkle Aftereffect

**FIGURE 12:**
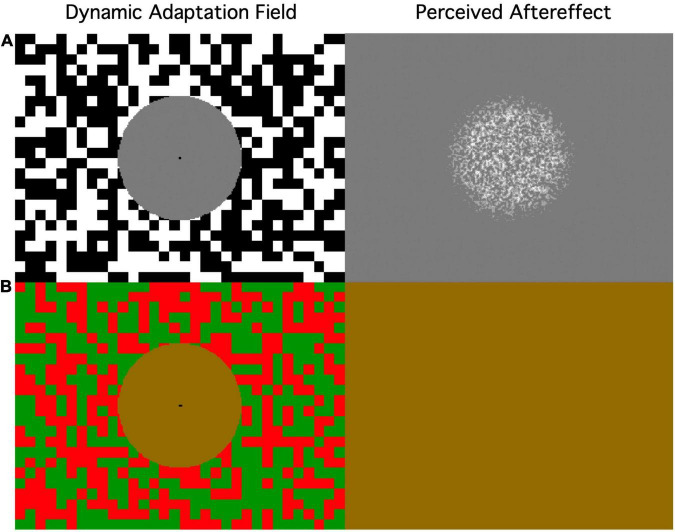
Depiction of the stimulus and aftereffect of the dynamic twinkle paradigm (From Tyler and Hardage, 1998). **(A)** Achromatic twinkle > 12 Hz. The aftereffect appears as twinkling noise in the *un*stimulated region (which does not exhibit filling in at this scale). **(B)** Purely chromatic twinkle evokes no aftereffect. This is a dynamic illusion of fiction of visual signals.

A further effect that generated dynamic aftereffects (though not with any form of motion direction) is the induced twinkle aftereffect (Hardage and Tyler, 1995), in which twinkling (dynamic) noise surrounding a gray field generates the aftereffect of a twinkling noise percept in the unstimulated gray region when it is turned off (Figure 12). Thus, it is an induced effect in the sense that it occurs in the unstimulated adjacent region, and an aftereffect in that it occurs after the stimulus offset. In both respects it is difficult to account for on Bayesian grounds.

A Bayesian form of explanation was evoked for a related effect reported by Ramachandran and Gregory (1991), in which a small gray patch inserted in the periphery of a field of dynamic noise was observed to fill in with similar dynamic noise after a few seconds of observation. They offered the interpretation that this was a filling-in process analogous to what is observed in the blind spot and in retinal scotomas, designed to achieve a continuous percept in the face of deficits in the retinal representation of the world. Moreover, when the noise field was removed, they observed the twinkling aftereffect in the unstimulated gray region, and proposed that this was the filling-in mechanism revealed by its slower time constant.

Tyler and Hardage (1998), however, tested the association between the two effects (the filling-in and the twinkle aftereffect) by varying the size of the gray field. They found that the filling-in was restricted to smaller field sizes and the aftereffect to larger field sizes (where filling-in was never observed), crossing over at just the size used by Ramachandran and Gregory. The implication of the dissociation is that the two effects are mediated by different processes, with the twinkle aftereffect being considered as resulting from a relativistic spatial normalization (or contrast effect) specific to the magnocellular pathway encoding dynamic fluctuations as neural signals that are sustained over time as a result of their characteristic rectifying non-linearity (Tyler and Hardage, 1998). This interpretation makes the prediction that, since chromatic signals are encoded by the linear parvocellular neurons, there should be no aftereffect for twinkling chromatic noise, which was found to be the case by Tyler and Hardage (1998).

Thus, while ***the filling-in process*** seems to be amenable to an adaptive explanation to match the Bayesian priors for fields of twinkling noise (Ramachandran and Gregory, 1991), the ***twinkling aftereffect*** in large unstimulated regions that were never perceived to fill in seems highly paradoxical in adaptive terms, and explainable only in terms of neural interconnectivity between the extended surround and center regions. In particular, the twinkling aftereffect is dissociated from classical spatial aftereffects in that it showed no dependence on the size of the inducing noise elements and always appeared to have the same spatial composition as size varied (This was in contrast to the filling-in percept, in which the perceived grain size varied with the grain size of the inducing noise; Hardage and Tyler, 1995).

## Perceptual Ambiguity at the Signal Level

### The Marroquin Textural Reorganization Effect

**FIGURE 13:**
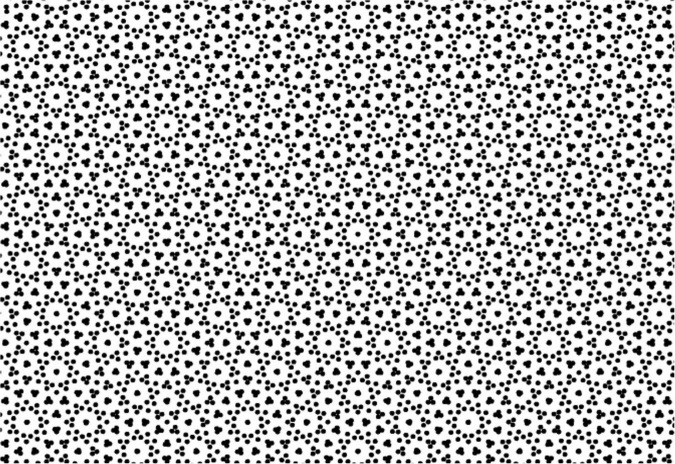
The multiply-ambiguous Marroquin texture (Marroquin, 1976). On sustained viewing, this texture spontaneously reorganizes into multifold Gestalt organizations, even with sustained fixation at one point, revealing the dynamic flux of perceptual analysis and hypothesis testing in a vivid fashion (See Tyler, 2021).

A final dynamic illusion that reveals the operation of the neural field processing system in an interesting manner is the Marroquin texture (Figure 13). This is a multistable texture that can be interpreted as having a large variety of Gestalt perceptual organizations (Marroquin, 1976). It should be observed steadily with minimal eye movements for about 30 s (but not so steadily as to be subject to stabilization fading). Unlike most other multistable Gestalt textures, however, each organization does not appear uniformly, but spreads capriciously across the pattern, usually beginning at the fovea. Thus, for example, if the organization of a particular small circle predominates, small circles are seen to pop into view at haphazard locations across the field for several seconds. A similar proliferation may be seen if the seed element is a larger circle, or a lenticular figure. If the organization switches to a cross configuration, similar crosses appear again across the image. Or a pattern of nested circles may expand out from the center.

Thus, the organization is seeded by the (Bayesian) foveal prior in a process that is slow relative to the perceptual observation time of the order of 100 ms, but extends across the field in what may best be characterized as a probabilistic serio-parallel fashion (in the sense that the spread of the prior across the field appears to be parallel, but the appearance of the percept at any given location appears to be probabilistically sequential rather than a uniform wave). The illusion that the elements are popping into view is an adventitious consequence of this probabilistic sequence. The fact that the structures fade back into a uniform texture after a second or two implies that they require an attentional component to become manifest.

The Marroquin effect clearly reveals the integrative processes of complex texture processing, and could be seen as a rather clunky form of Bayesian adaptive processing, at least given that the foveal information provides an appropriate prior for the rest of the visual field. Note that this processing is similar to what is generally needed for object processing, which is to link together different elements that form a coherent object, except that here the similar elements form a uniform texture. The fact that the elements can form star-shaped and expanding circular figures suggests that the linking system incorporates many forms of symmetry, not just the translational symmetry of the small circular elements.

**FIGURE 14:**
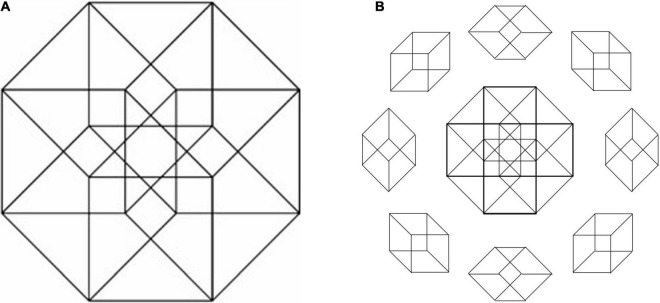
**(A)** Orthographic projection of a 4D hypercube to a 2D figure evokes multiply ambiguous 3D percepts of interconnected cubes. **(B)** Flattened version of **(A)** induced by minor changes to the configuration, surrounded by the 8 possible individual cube structures visible in **(A)**.

A similar degree of multiply ambiguous popout expanded to three dimensions is seen in the axonometric projection of the 4D hypercube (Tyler, 2021) as depicted in Figure 14A. It is almost impossible to see this as a flat configuration (like the center image of Figure 14B). Instead, the various cubes of which it is composed, shown around the circle in Figure 14B, pop in and out in a random succession of 3D interpretations.

## Five-Fold Devil’s Pitchfork

**FIGURE 15:**
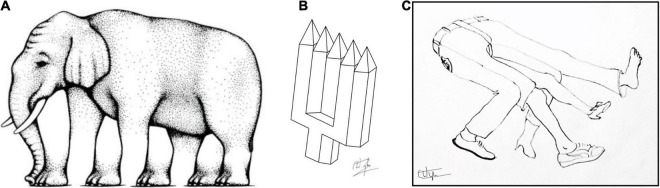
**(A)** Roger Shepard’s ambiguous-footed elephant. **(B)** My own five-tined devil’s pitchfork (or polivet). **(C)** My five-legged jeans version. All are illusions of multiple ambiguity.

Shepard’s Elephant, in which four legs morph into five (Figure 15A), may be generalized in cases such as the well known Devil’s Pitchfork illusion, in which two prongs morph into three. My own version (Figure 15B), was able to amplify the effect to achieve a morphing from only two prongs into five (i.e., three extra prongs, not just one). This notion can be applied to organic forms to provide a bizarrely amusing effect (Figure 15C).

## Overview

The important aspect of this series of illusions as that they each embody the ability to verify the non-illusory state, mostly by virtue of being printed on a static page that can be verified by inspection of the material by touch and by visual examination from many viewpoints. (a consequence that would be more difficult to verify if they were presented electronically). Thus, this survey validates the concept of illusions as percepts that can be ***directly verified*** by sensory means as deviations from the ground truth by operations available to the viewer (as opposed to relying on unverifiable assertions by experts), in the manner proposed by Ebbinghaus (1913) and subsequent authors. This constraint is intended to exclude verification by equipment other the same sensory apparatus that is viewing the illusion.

Thus, the perceptual identity of pairs of color metamers is not considered to be an illusion by this criterion, because there is no sensory means to verify that the two colors have different wavelength distributions, even though their spectrograms can be shown to be different if the requisite spectroscopy equipment is available to test them. Since most of the illusions discussed here provide novel impressions that are never seen outside the laboratory, it seems implausible that any of the three levels of priors, evolutionary, ontogenetic, or short-term adaptive, are of much relevance to the perceptual interpretations described here. It may therefore be proposed that they reveal the adventitious operation of the perceptual mechanisms recruited over the short evolutionary history of the human brain, which did not include the variety of visual tasks facing our modern world. In other words, these illusions are maladaptive under the conditions of the illusion, creating an interpretation of reality that diverges from the verifiable construction—straight lines appear curved, fictional structures are generated with no supporting evidence, single structures have multiple sequential interpretations, and static displays appear dynamic.

It is possible to generate plausible adaptive explanations for some of these effects in different contexts, although that will not be attempted here because they smack too much of Greek-God-style origin fables. Instead, the focus is on the maladaptive nature of the perceptual processing under the specific contexts of the illusions, which, though unusual, might still have been encountered in everyday life in some form. Most illusions seem to fall under the general rule of contextual surround effects, where the target of attention is seen as distorted in some biasing context, relative to how it appears in isolation. Such effects are explained by the particular trick from the perceptual bag-of-tricks of local normalization relative to the surrounding context, which is a general principle of neural processing operating at all levels, from the earliest levels of retinal processing to the highest conceptual levels of not just object processing but abstract conceptualization such as principles of government. Such spatiotemporal context effects account for not only contrast effects in many perceptual domains, but the wide range of perceptual constancies, figural salience, attentional targeting, and the linguistic and philosophic sense of the meaning of any linguistic utterance.

In one sense, this contextual view of perceptual illusions is adaptive, in that it adapts a convenient principle of neural organization to economical neural coding at many levels of operation. But in the specific sense it is maladaptive, since this efficient coding leaves numerous pockets of coding errors that we call “illusions” because the deviate from the verifiable ground truth of the situation without of the context that is inducing the illusion.

## Conclusion

The present range of 2D and 3D illusory percepts directly observable by the reader is designed to amplify the point that there is a wide variety of percepts that cannot straightforwardly be considered to be an interpretation of the stimulus array (which in cases of adaptational illusions can be a uniform test field with no discernable visual information at all). Such percepts require a category of perception to describe them, for which the term “perceptual illusions” seems entirely appropriate. In many cases, particularly those that generate a sense of motion from observation of a static field, pose interesting challenges to an explanatory framework for perception, both at the physiological level of underlying neuronal circuitry and at the conceptual level of Bayesian or evolutionary explanations. Even after two centuries of investigation, illusions retain their fascination as probes of the nature of perceptual processing.

## Data Availability Statement

Publicly available datasets were analyzed in this study. This data can be found here: What are publicly available are the demonstrations provided in the figures, which provide the self-generated data from each reader.

## Author Contributions

The author confirms being the sole contributor of this work and has approved it for publication.

## Conflict of Interest

The author declares that the research was conducted in the absence of any commercial or financial relationships that could be construed as a potential conflict of interest.

## Publisher’s Note

All claims expressed in this article are solely those of the authors and do not necessarily represent those of their affiliated organizations, or those of the publisher, the editors and the reviewers. Any product that may be evaluated in this article, or claim that may be made by its manufacturer, is not guaranteed or endorsed by the publisher.
